# Oral Hygiene

**Published:** 1892-06

**Authors:** J. W. Wassall

**Affiliations:** Chicago, Ill.


ARTICLE II.

### ORAL HYGIENE.

BY J. W. WASSALL, M. D., D. D. S., CHICAGO, ILL.

### A Plea for the Prevention of Dental Diseases by the Establishment of Proper Habits of Cleanliness.

All statistics of civilized communities prove a gradual amelioration and prolongation of human life.

Mr. George H. Knight, in an article published in the February number of the *Cosmopolitan*, makes the pertinent statement that for the decade ending 1860, A. D., the annual death rate for New York city was 33.66 per thousand. For the semi-decade ending 1865 it had fallen to 31.33 and in the semi-decade ending 1890 to 25.54; the rate for 1890 being 24.58. This amounts to a saving in the city mentioned of 3,000 lives annually. These results are attributed to the advance of medical science and of state and personal hygiene.

The same writer has it that the average life in Great Britain is nine years longer than it was fifty years ago.

It was the knowledge of these or similar authentic statistics, no doubt, which led Dr. J. Y. Crawford, at the recent banquet given to the Executive Committee of the World's Columbia Dental Congress—rather too warmly, perhaps—to claim that modern dental science had exerted greater potency than all other causes in bringing about this felicitous result. No intelligent observer will deny that the great awakening of civilized men, particularly noticeable in the States, as to the importance of preserving the natural teeth, has in the last half century, been a perceptible influence in bringing about the increased longevity alluded to. The profession of dentistry then bears a more important relation to human progress and comfort than is usually admitted even by the profession itself. To realize the truth of this statement one has but to picture to himself the unequal odds in the struggle for life, of a community without services of conservative dentistry, say for four generations. In other words the proposition is tenable that a man with defective teeth is to that extent unfitted to fulfill the requirements of human life and activity.

Medical science, which, in its broadest sense includes the practice of dentistry, has two purposes—restoration of the abnormal to normal, and the prevention of the abnormal. We have to do tonight with the latter question, and the task to which I have set myself is to endeavor to bring out, in some small measure, the part to be played by prophylactic dentistry in the department of preventive medicine.

The scientific and modern practice of dentistry implies that along with the restorative measures applied by dental surgery to the correction of lesions of the teeth, must go the combative or preventive measures supplied by the observance of rules of oral hygiene.

Generally speaking, the whole range of pathological conditions to which the teeth are subject, may be classified under two heads:

1. Diseases affecting the crowns of the teeth.

2. Disease affecting the roots and socket. Except traumatic lesions, both these classes of disease are preventable by conscientiously following strict rules of hygiene. An attempt will be made later on to formulate these rules.

It requires no argument to persuade you of the truth of the hypothesis that "the disease known as dental caries will not occur except where the causal microbes are permitted to grow undisturbed upon the teeth." The rational conclusion is then, that if the teeth are not allowed to accumulate deposits upon either their exposed or protected surfaces, they will be exempt from caries. More broadly stated the proposition is:

Given the varying predispositions of different individuals to caries, which is governed by the laws of heredity and environment, the growth of micro-organisms in the mouth is in proportion to the amount of disturbance they suffer, or, rest and opportunity they enjoy. That is a recognized fact, and almost axiomatic, I believe.

Is the practice of dentists as followed in the daily routine of seeing patients consistent with these undisputed etiological facts? We all know it is not.

Plainly, then, one great step in the direction of the establishing of correct habits of cleansing the teeth in the general public is to be accomplished by reform of the dentist, making obligatory upon him the performance of his duty. Similarly, as public sentiment now requires a man to be a graduate of a reputable college before entering upon practice, so can it compel him to teach oral hygiene and require his patients to observe its rules.

For the purpose of convenience, allow me to place people in three classes with respect to the care they give their teeth: Let us say that a certain number of people —the figures do not pretend to be accurate—by assiduous, intelligent care and a natural tendency to cleanliness in the teeth themselves, have absolutely clean teeth from year to year; the proportion is very small indeed, say one per cent. A second class are just as anxious and spend as much time, or perhaps more, than class one, but their efforts are ineffective because misdirected and on account of unsuitable cleansing implements and materials. This class constitutes say nine per cent. All the others who give slovenly or no care, belong to class three, forming the largest proportion, namely, ninety per cent.

If we are justified in assuming that caries and pyorrhœa alveolaris, the two diseases most destructive to the teeth, only occur in the presence of foreign matter which is allowed to accumulate upon them, we next want to know how to prevent the accumulations.

My own belief is, that it is possible in most cases for the individual to keep the teeth absolutely free from all deposits and food debris. I have been led to this belief by the study of several cases which have been under my care and observation for some time past.

The following would be the way of dealing with a person belonging to Class II or III who make periodical visits to have the mouth put in order.

At the first appointment, examine the mouth as to the degree of cleanliness usual to the person, and make a note of the conditions in the record book for future reference. Catechize the person to ascertain what are the present habits as to the number of brushings per diem, dentifrices, floss, tooth-pick, etc. Make notes of each point. Then show to the person with mirrow every part of the teeth which is unclean. The patient may apologetically, or even indignantly say that it is impossible to be more thorough than he is. It is not prudent to deny this at the time if you wish to accomplish your purpose, unless to explain that you may be able to help him to be more successful. You must gain his good will and inspire the desire by giving arguments for ordinary (ordinary in its new sense) cleanliness and explaining its benefits.

It is now proper to scale and polish perfectly each tooth. The mirrow should now be used again to show the patient the change. Now is your time to teach a lesson of the most forcible kind. Say to him, "it is quite possible for you, by your own efforts, to keep every tooth in your mouth absolutely clean." Make this statement positively and dogmatically. With the effrontery, if you please of a bichloride of gold doser promising his dupe a sure cure. The moral effect of such an assurance and the placing of the responsibility where it belongs will be very helpful.

The instructions in the daily care to be given the teeth should now be distinctly and carefully impressed upon the patient's mind. Printed directions for children might be useful but I have never tried them.

Now, you may say all this and more—some people will acquire the habit easily—others, never. It is your business to labor patiently to the right end.

At the next appointment, for a filling, perhaps, make inquiries to see that your instructions have been followed.

If they have not, in every detail, reassert the necessity for it, refreshing the patient's mind, and, in some cases, demonstrate with a brush the practicability of your statements. Be careful not to lay down more rules than can be followed.

This system of following up the matter as long as the appointment lasts, seeing to it that the patient's efforts are sustained and effective and that the habit is formed, are the most important and valuable services you can render in your capacity as a dentist.

The task is not a thankless one; your labor will invariably be highly appreciated, and as these patients come back to you from time to time, it will be one of the greatest satisfactions of your professional experience to be able to say, after the most conscientious examination with the most searching of fine explorers, that you find no decay—no pockets

Now as to rules: Of first importance, of course, is the use of the brush. Twice daily is sufficient—at night before retiring and in the morning. It is of the utmost advantage to have three brushes in use. This is imperatively required—*three brushes.* A brush will not do effective work unless it has time to dry out. The bristles will always be too soft if it is used more than once in twenty-four hours. More good is obtained by this than one would expect without trying. What is known to the dealers as "medium" grade of stiffness is the best. To the well kept mouth with all its parts and members in a healthy state, a good vigorous brushing with a moderately stiff brush is a pleasurable sensation. Three brushes in use at the same time will also give longer service than if bought consecutively.

The usual instructions as to vertical movements should be given. Special directions are necessary to make the patient reach all the accessible surface. Remember that few people do this. It becomes your duty to dispel the common delusion that the last molar and lingual surfaces cannot be brushed. Anyone can touch all these surfaces who will use his brain, and for a time watch himself during the process. The old slovenly or thoughtless habit is to be broken up and a new set of movements learned. Insist on thoroughness, and keep on insisting.

Dentifrices: Some form of powder is the only proper dentifrice and it should always be used whenever the brush is used.

A pound can or bottle from which the small bottle for present use may be replenished is indispensable in preventing a giving out of the supply and a consequent deposit of calculus by a few days' forgetfulness to go to the druggist. Aim to make the powder as agreeable as possible, especially for children.

Women and children can be induced to use floss silk or rubber bands for interdental spaces at least once a day. Men should be encouraged to use the tooth-pick.

Disinfectant and antiseptic washes are allowable and useful, but should not take the place of powder applied by brush at the stated intervals. As a rule I recommend it only in special cases. The best results are obtained by the simplest means faithfully employed. It is more than probable that the individual whom we have in hand will have anywhere from three to a dozen appointments for the other needed operations. Opportunities are thus afforded to assist in putting him on the right track. You have given him a good start—how shall he be watched?

One or two months later, by previous arrangement, he must be summoned to your office for inspection. Perhaps—yes usually—a little scolding and lecturing will be necessary to put him right. At suitable intervals he must be notified again and both of you will be delighted at the fine appearance and freedom from disease presented.

My own experience is that it is not a difficult matter to bring about this happy condition and one success pays for a score of failures.

### RESUME.

A statement to the patient of the advantages and desirability of absolute cleanliness of the teeth.

Namely, that it affords immunity from caries of crown and loosening of tooth in socket.

**Economy of time.**

**Economy of money.**

**Economy of pain, and preservation of natural teeth all through life.**

**A statement of the means to be employed to maintain absolute cleanliness of the teeth.**

**Establishment of habit.**

**Three brushes for daily use.**

Thorough brushing of labial and lingual surfaces twice daily with powder.

Silk, rubber bands or pick.

Regular visitation by appointments for inspection and instruction.—*Dental Review.*

